# Retraction: Postoperative pulmonary complications in patients with transcatheter tricuspid valve implantation—implications for physiotherapists

**DOI:** 10.3389/fcvm.2025.1563660

**Published:** 2025-01-24

**Authors:** Frontiers Editorial Office

A Retraction of the Original Research Article Postoperative pulmonary complications in patients with transcatheter tricuspid valve implantation—implications for physiotherapists By Yu P-M, Wang Y-Q, Luo Z-R, Tsang RCC, Tronstad O, Shi J, Guo Y-Q and Jones AYM (2022). Front. Cardiovasc. Med. 9:904961. doi: 10.3389/fcvm.2022.904961

The journal retracts the 19 May 2022 article cited above.

Following concerns regarding the originality of the article, an investigation was conducted in accordance with Frontiers' policies. During the course of this investigation, Frontiers attempted to reach the Biomedical Ethics Committee of West China Hospital of Sichuan University for more information however, they could not be reached. The investigation found that the complaint was valid and that the article should be retracted due to an unacceptable level of similarity to the article:

Wang Yuqiang, Shi Jun, Liu Lulu, Luo Zeruxin, Zhang Fengmei, Guo Yingqiang, Yu Pengming. The initial exploration of pulmonary complications and rehabilitation recommendations after transcatheter tricuspid valve intervention: a prospective cohort study. *Chinese Thoracic and Cardiovascular Surgery Clinical Journal*, 2022, 29(8): 963–970. doi: 10.7507/1007-4848.202111038.

This retraction was approved by the Chief Editor of Frontiers in Cardiovascular Medicine: Heart Surgery and the Chief Executive Editor of Frontiers. The authors did not reach an agreement on this retraction due to some authors being absent on the author list of the publication with The Chinese Thoracic and Cardiovascular Surgery Clinical Journal.

We would like to note that Dr Alice Jones, Dr Raymound Tang, and Dr Oystein Tronstad did not appear on the author list for the publication in the Chinese Thoracic and Cardiovascular Surgery Clinical Journal, and that they stated they were unaware of this publication.

